# Parental Attitudes Toward Childhood Vaccination in Riyadh, Saudi Arabia: A Cross-Sectional Study

**DOI:** 10.7759/cureus.107631

**Published:** 2026-04-24

**Authors:** Amani M Nabri, Deema Aljasser, Asma AlMutairi, Mohammed Bokir, Reema M Alshehri, Rawan Alhammadi, Rian Balafkhar, Lubna Alnosyan, Abdullah M Abu Qarnayn, Somia A Mousa, Sara AlGhamdi

**Affiliations:** 1 Clinical Sciences, Dar Al Uloom University, Riyadh, SAU; 2 Family Medicine, Dar Al Uloom University, Riyadh, SAU; 3 Medicine, Dar Al Uloom University, Riyadh, SAU; 4 College of Medicine, Alfaisal University, Riyadh, SAU; 5 College of Medicine, King Khalid University, Abha, SAU; 6 College of Medicine, Dar Al Uloom University, Riyadh, SAU; 7 Medicine and Surgery, Almaarefa University, Riyadh, SAU

**Keywords:** childhood immunization, childhood vaccination, health politics, parental attitudes, pediatrics

## Abstract

Background

Parental attitudes toward childhood vaccination play a critical role in vaccine uptake. Several factors can influence parental attitudes regarding vaccination. This study aimed to assess parental attitudes toward childhood vaccination in Riyadh, Saudi Arabia, and examine the influence of sociodemographic factors.

Methodology

A cross-sectional study was conducted among parents residing in Riyadh using an online, self-administered questionnaire distributed via social media platforms. The survey instrument was based on the Vaccine Attitudes Examination scale and consisted of 11 Likert-scale items. Data were analyzed using SPSS version 28.

Results

A total of 469 parents participated. Most respondents were female (n = 286, 61.0%), aged 25-44 years (n = 257, 54.8%), married (n = 402, 85.7%), and held a bachelor’s degree (n = 267, 56.9%). Positive perceptions of vaccination were common, as 58.5% (n = 274) agreed or strongly agreed that they felt safe after vaccination, and 65.7% (n = 308) agreed or strongly agreed that vaccines prevent serious infectious diseases. Most participants also valued reliable information sources, with 81.2% (n = 381) agreeing or strongly agreeing that accurate information supports informed decisions. However, concerns persisted regarding unforeseen side effects and beliefs favoring natural immunity over vaccination. Overall, most participants demonstrated neutral attitudes (n = 348, 74.2%), followed by negative attitudes (n = 83, 17.7%) and positive attitudes (n = 38, 8.1%). Gender (p = 0.022) and employment status (p = 0.032) were significantly associated with attitudes. In multivariable analysis, female gender (odds ratio (OR) = 2.113, 95% confidence interval (CI) = 1.227-3.640, p = 0.007), larger family size (OR = 1.538, 95% CI = 1.043-2.269, p = 0.030), and employment status (OR = 1.299, 95% CI = 1.005-1.680, p = 0.046) significantly predicted negative attitudes.

Conclusions

Most parents in Riyadh demonstrated neutral attitudes toward childhood vaccination, while nearly one in five showed negative attitudes. Targeted educational strategies addressing safety concerns and misinformation may help strengthen vaccine confidence among parents.

## Introduction

Vaccination remains the most effective public health intervention in children to reduce morbidity and mortality associated with infectious diseases. Routine immunization programs have successfully contributed to the control and eradication of various critical illnesses such as measles, poliomyelitis, and diphtheria [1]. Despite these efforts, vaccine hesitancy has also emerged as a growing concern. The World Health Organization has identified vaccine hesitancy as one of the top global health threats [2]. Parental attitude also influences childhood vaccination uptake, as parents are the primary decision makers for the health of children; therefore, their beliefs, perceptions, and concerns directly affect immunization compliance [3]. Positive attitudes of parents are associated with higher vaccination rates, whereas negative perceptions about vaccination can lead to vaccine hesitancy. Several factors influence these perceptions, including individual, social, and systemic factors such as education, cultural beliefs, access to healthcare, and exposure to reliable health information [4,5]. Another factor in this regard is political trust. Trust in authorities influences how individuals perceive health recommendations, interpret risk, and respond to public health campaigns [6]. When trust in authorities is high, individuals are more likely to accept vaccination as a credible and necessary preventive measure.

Saudi Arabia has made substantial progress in implementing comprehensive childhood immunization programs [7]. The country’s healthcare system provides free access to vaccinations, supported by structured national policies and awareness campaigns led by the Ministry of Health, Saudi Arabia, with more than 90% of school-going children completely immunized [8]. However, like many countries, Saudi Arabia is not immune to the challenges posed by vaccine hesitancy. For example, a systematic review from Saudi Arabia reported that vaccine hesitancy in Saudi Arabia ranged from 6.5% to 80%, with a total prevalence of 41.6% [9]. The study further concluded that several factors can reduce vaccine hesitancy, including media campaigns, leaders involved in the vaccination process, trust in medical practitioners, free vaccinations, and religion. Similar other studies have shown that concerns related to vaccine safety, long-term effects, and perceived external influences such as political or financial motives are key factors in vaccine hesitancy [10]. Gentile and Alesi found that the parents of children who distrust science and believe in conspiracy theories are hesitant to vaccinate [11].

International evidence has also demonstrated that attitudes toward vaccination can be influenced by communication and external messaging. For example, a cross-sectional survey of 411 parents in Australia found that 76.2% of participants had non-fixed views regarding vaccination, while 23.8% held fixed views, including 21.7% pro-vaccination and 2.2% anti-vaccination. Parents with non-fixed views were more likely to change their willingness to vaccinate after exposure to vaccine-related messages and were also more vulnerable to increased hesitancy after negative messaging [12]. These findings highlight the importance of understanding parental attitudes within specific communities, as views may shift depending on the information environment and level of trust in health recommendations. Riyadh, as the capital city and one of the largest urban centers in Saudi Arabia, provides an important setting for evaluating parental attitudes toward childhood vaccination. Parents in Riyadh may benefit from better access to healthcare facilities and educational resources, yet they may also encounter misinformation through social media and informal communication channels. Assessing parental attitudes in this setting can provide valuable insight into the factors that promote confidence in vaccination or contribute to hesitancy.

Therefore, this study aims to assess parental attitudes toward childhood vaccination in Riyadh, Saudi Arabia, and to explore factors associated with these attitudes.

## Materials and methods

Study design and setting

This cross-sectional study was conducted to assess parental attitudes toward childhood vaccination in Riyadh, Saudi Arabia. The study was conducted among Saudi nationals and residents living in Riyadh. Data collection was performed via online platforms. Participants were contacted through social media applications such as WhatsApp and Telegram and invited to complete a Google Forms questionnaire.

Study population and participants

The study population consisted of parents, including both mothers and fathers, residing in Riyadh, Saudi Arabia. Inclusion criteria included Saudi nationals and residents who were parents and willing to participate in the study. The required sample size was calculated using a standard formula for cross-sectional studies, assuming a population size (N) of 7,953,000, a confidence level of 95% (Z = 1.96), a margin of error of 5% (E = 0.05), and a proportion (p) of 0.5 to ensure maximum sample size [13]. Based on these parameters, the minimum required sample size was 384 participants.

Sampling technique and data collection

A convenience sampling method was used in this study. The questionnaire link was distributed online through social media platforms. Data were collected using a structured online questionnaire developed through Google Forms. The instrument was based on the Vaccine Attitudes Examination scale [14] and consisted of 11 items measured on a Likert scale ranging from strongly disagree to strongly agree. The questionnaire assessed participants’ perceptions, beliefs, and attitudes toward childhood vaccination. The total attitude score was calculated by summing responses across all 11 Likert-scale items. To standardize interpretation, the total score was converted into a percentage of the maximum possible score. Based on this percentage, participants were categorized into the following three groups: negative attitude (<50%), neutral attitude (50-75%), and positive attitude (>75%) toward childhood vaccination. Binary logistic regression was performed to identify predictors of negative attitudes.

Internal consistency of the 11-item questionnaire was assessed using Cronbach’s alpha. Inter-item correlations were examined to evaluate the relationship among items, and items with reverse wording were recoded where necessary before analysis. Exploratory factor analysis was performed to examine the underlying factor structure of the scale. Sampling adequacy was checked using the Kaiser-Meyer-Olkin (KMO) test, and Bartlett’s test of sphericity was used to confirm that the data were suitable for factor analysis. The questionnaire showed acceptable sampling adequacy (KMO = 0.814), and Bartlett’s test of sphericity was significant (χ² = 2952.156, df = 55, p < 0.001), supporting factor analysis. The overall Cronbach’s alpha was 0.664, indicating moderate internal consistency.

Ethical considerations

Ethical approval was obtained from the Institutional Review Board (IRB) of Dar Al Uloom University before conducting the study (approval number: HP-01-R-134- DAU-COM-26-11). The study adhered to the principles of the Helsinki Declaration. Participants were informed about the purpose of the study, and confidentiality and anonymity were strictly maintained. Participation was voluntary, and data were used solely for research purposes.

Statistical analysis

Data were analyzed using SPSS version 28.0 (IBM Corp., Armonk, NY, USA), and Microsoft Excel 2010 (Microsoft Corp., Redmond, WA, USA) was used for data management and processing. Descriptive statistics were used to summarize the data. Inferential statistical analysis was performed using the chi-square test to assess associations between categorical variables. A p-value of less than 0.05 was considered statistically significant.

## Results

A total of 469 participants were included in the study. Most respondents were aged 25-34 years (n = 128, 27.3%) and 35-44 years (n = 129, 27.5%), followed by those aged 45-54 years (n = 105, 22.4%). Regarding sex, females constituted the majority (n = 286, 61.0%), whereas males accounted for 183 (39.0%) participants. Most participants were married (n = 402, 85.7%), while 46 (9.8%) were divorced and 21 (4.5%) were widowed. Regarding education, over half of the respondents held a bachelor’s degree (n = 267, 56.9%). Most respondents lived in urban areas (n = 406, 86.6%), while 63 (13.4%) resided in suburban locations. Nearly half of the participants reported having four to six family members (n = 234, 49.9%), followed by one to three members (n = 142, 30.3%). Regarding employment status, more than half were employed full-time (n = 257, 54.8%). Additionally, 104 (22.2%) were unemployed, and 44 (9.4%) were retired. Table 1 shows the sociodemographic characteristics of the study participants.

**Table 1:** Sociodemographic characteristics of the study participants (N = 469).

		N	%
Sex	Male	183	39.0
	Female	286	61.0
Marital status	Divorced	46	9.8
	Married	402	85.7
	Widowed	21	4.5
Education	Bachelor’s degree	267	56.9
	Diploma	57	12.2
	Doctorate or higher	20	4.3
	High school or less	63	13.4
	Master’s degree	62	13.2
Geographical location	Suburban	63	13.4
	Urban	406	86.6
Family members	1–3	142	30.3
	4–6	234	49.9
	6 or more	93	19.8
Employment status	Retired	44	9.4
	Unemployed	104	22.2
	Employed full-time	257	54.8
	Employed part-time	40	8.5
	Others	24	5.1

Table 2 shows the responses of the participants to questions related to their attitude toward vaccination. A large proportion of respondents expressed positive perceptions regarding vaccine safety and effectiveness. For instance, most participants agreed or strongly agreed that they feel safe after being vaccinated (n = 141, 30.1%; n = 133, 28.4%) and that vaccines can prevent serious infectious diseases (n = 157, 33.5%; n = 151, 32.2%). Similarly, many respondents somewhat agreed that they feel protected after vaccination (n = 131, 27.9%); however, a significant proportion expressed some level of disagreement (n = 98, 20.9%; n = 101, 21.5%). Concerns and skepticism about vaccines were also evident. A considerable number of participants strongly disagreed or disagreed with statements suggesting hidden political motives behind vaccines (n = 148, 31.6%; n = 101, 21.5%). Similarly, many respondents rejected the idea that vaccines primarily benefit pharmaceutical companies rather than the public (n = 202, 43.1%; n = 132, 28.1%) and disagreed that authorities promote vaccination for financial gain (n = 231, 49.3%; n = 108, 23.0%). However, some uncertainty remained regarding vaccine safety. A proportion of participants somewhat agreed that vaccines may cause unforeseen problems in children (n = 130, 27.7%) and expressed concerns about unknown future effects. Furthermore, mixed opinions were observed regarding misconceptions about vaccination. A substantial proportion agreed or somewhat agreed that vaccination programs may serve national agendas (n = 157, 33.5%; n = 144, 30.7%) and that natural immunity may last longer than vaccination (n = 162, 34.5%; n = 115, 24.5%). Most participants agreed or strongly agreed that access to accurate information helps in making optimal decisions about childhood vaccination (n = 139, 29.6%; n = 242, 51.6%).

**Table 2:** Participants’ attitudes toward vaccination (N = 469).

Questions	Strongly disagree	Disagree	Somewhat disagree	Somewhat agree	Agree	Strongly agree
I feel safe after being vaccinated	31 (6.6)	22 (4.7)	34 (7.2)	108 (23.0)	141 (30.1)	133 (28.4)
I can rely on vaccines to stop serious infectious diseases	29 (6.2)	12 (2.6)	22 (4.7)	98 (20.9)	157 (33.5)	151 (32.2)
I feel protected after getting vaccinated	54 (11.5)	98 (20.9)	101 (21.5)	131 (27.9)	56 (11.9)	29 (6.2)
Although most vaccines appear to be safe, there may be tampering to serve specific political matters that we have not yet discovered	148 (31.6)	101 (21.5)	67 (14.3)	82 (17.5)	42 (9.0)	29 (6.2)
Vaccines can cause unforeseen problems in children	36 (7.7)	83 (17.7)	104 (22.2)	130 (27.7)	71 (15.1)	45 (9.6)
I worry about the unknown effects of vaccines in the future	155 (33.0)	144 (30.7)	77 (16.4)	61 (13.0)	20 (4.3)	12 (2.6)
Vaccines make a lot of money for pharmaceutical companies and countries, but do not do much for regular people	202 (43.1)	132 (28.1)	51 (10.9)	54 (11.5)	19 (4.1)	11 (2.3)
Authorities promote vaccination for financial gain, not for people’s health	231 (49.3)	108 (23.0)	48 (10.2)	47 (10.0)	22 (4.7)	13 (2.8)
Vaccination programs are a big con to serve the country’s agenda	26 (5.5)	33 (7.0)	54 (11.5)	157 (33.5)	144 (30.7)	55 (11.7)
Natural immunity lasts longer than vaccination	27 (5.8)	31 (6.6)	53 (11.3)	115 (24.5)	162 (34.5)	81 (17.3)
Access to accurate information from reliable sources helps people reach optimal decisions about the importance of childhood vaccination	12 (2.6)	17 (3.6)	22 (4.7)	37 (7.9)	139 (29.6)	242 (51.6)

**Figure 1:**
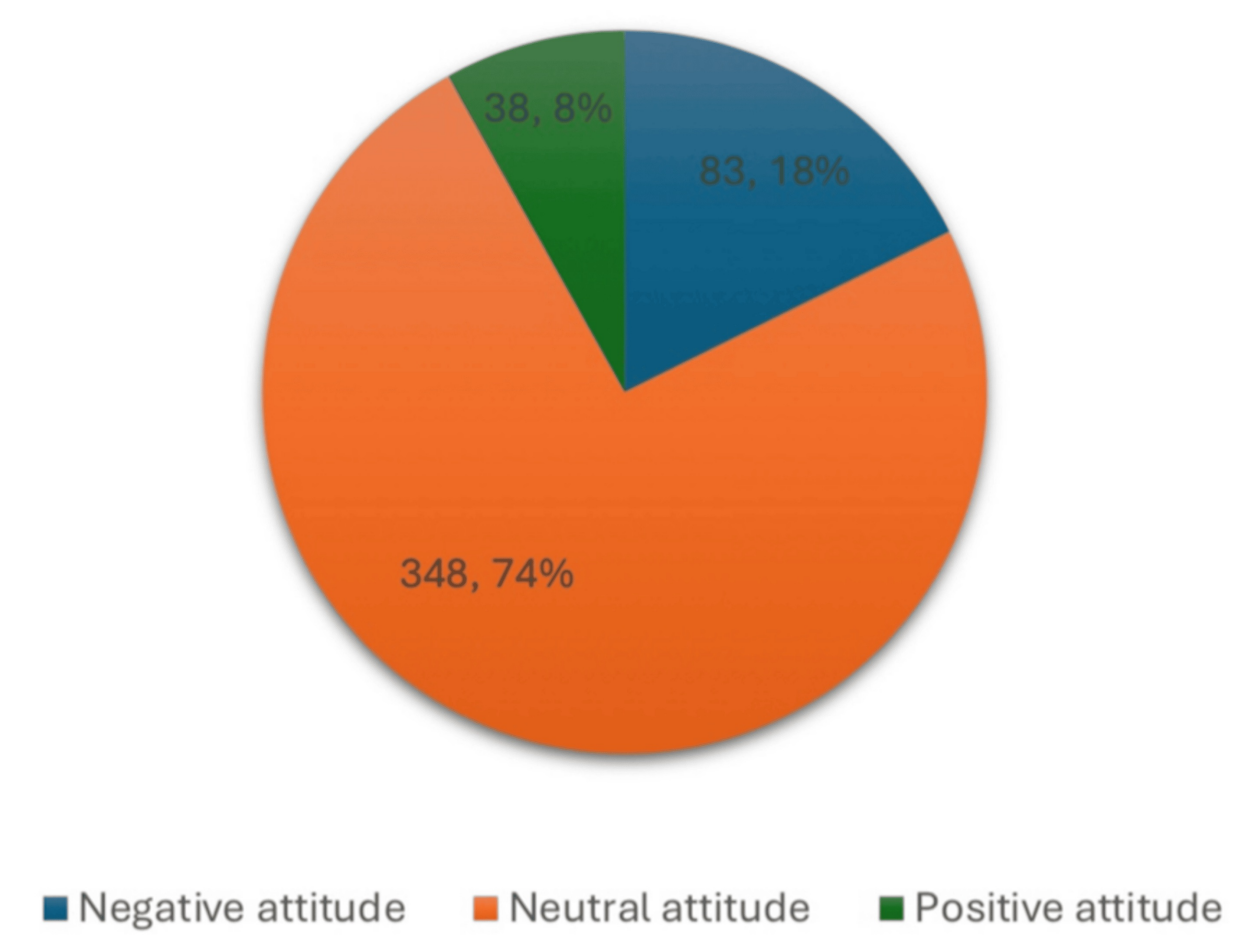
Parental attitudes toward childhood vaccination (N = 469).

Figure 1 shows parental attitudes toward childhood vaccination. Most participants exhibited a neutral attitude (n = 348, 74.2%). Negative attitudes were observed among 83 (17.7%) respondents, while only a small proportion demonstrated positive attitudes (n = 38, 8.1%).

Table 3 shows the association between sociodemographic variables and parental attitudes toward childhood vaccination. With respect to geographical location, the majority of both suburban (n = 50) and urban residents (n = 298) showed neutral attitudes with no statistically significant association observed (p = 0.294). Similarly, marital status did not show a significant relationship with attitudes (p = 0.232). Age groups also did not demonstrate a statistically significant association with vaccination attitudes (p = 0.381). Sex, however, showed a statistically significant association with attitudes toward vaccination (p = 0.022). A higher proportion of females exhibited neutral (n = 200) and negative attitudes (n = 61) compared to males, while positive attitudes were relatively low in both groups. Educational level was not significantly associated with attitudes (p = 0.228). Similarly, family size showed no significant association (p = 0.119). In contrast, employment status demonstrated a statistically significant association with vaccination attitudes (p = 0.032).

**Table 3:** Association between sociodemographic characteristics and parental attitudes toward childhood vaccination (N = 469). *: significant p-values.

Variable	Category	Negative	Neutral	Positive	P-value	χ²
Geographical location	Suburban	11	50	2	0.294	2.44
	Urban	72	298	36		
Marital status	Divorced	7	38	1	0.232	5.59
	Married	73	292	37		
	Widowed	3	18	0		
Age (years)	18–24	7	23	4	0.381	10.70
	25–34	16	101	11		
	35–44	23	96	10		
	45–54	23	77	5		
	55–64	10	37	8		
	65 or above	4	14	0		
Sex	Male	22	148	13	0.022*	7.63
	Female	61	200	25		
Education	Bachelor’s degree	43	199	25	0.228	10.56
	Diploma	11	46	0		
	Doctorate or higher	4	15	1		
	High school or less	15	40	8		
	Master’s degree	10	48	4		
Family members	1–3	20	110	12	0.119	7.34
	4–6	38	178	18		
	6 or more	25	60	8		
Employment status	Retired	6	35	3	0.032*	16.82
	Unemployed	24	70	10		
	Employed full-time	36	202	19		
	Employed part-time	8	30	2		
	Others	9	11	4		

Binary logistic regression was conducted to identify predictors of negative attitude (1 = negative attitude; 0 = neutral/positive attitude). The overall model was statistically significant (Omnibus χ² = 16.811, df = 7, p = 0.019), indicating that the included variables significantly improved the prediction of negative attitude. The model demonstrated acceptable fit according to the Hosmer-Lemeshow test (χ² = 13.582, df = 8, p = 0.093). The explained variance was modest (Cox and Snell R² = 0.035; Nagelkerke R² = 0.058). Sex was the strongest significant predictor of negative attitude (odds ratio (OR) = 2.113, 95% confidence interval (CI) = 1.227-3.640, p = 0.007), followed by family members (OR = 1.538, 95% CI = 1.043-2.269, p = 0.030) and employment status (OR = 1.299, 95% CI = 1.005-1.680, p = 0.046). Age, marital status, education, and geographical location were not significantly associated with negative attitudes (Table 4).

**Table 4:** Binary logistic regression analysis of factors associated with negative attitude. OR: odds ratio; CI: confidence interval

Predictor	B	Wald	P-value	OR (Exp(B))	95% CI for OR	Interpretation
Age	0.063	0.328	0.567	1.065	0.858–1.322	Not significant
Sex	0.748	7.276	0.007	2.113	1.227–3.640	Significant
Marital status	-0.154	0.206	0.650	0.857	0.440–1.669	Not significant
Education	0.054	0.446	0.504	1.055	0.901–1.237	Not significant
Geographical location	-0.008	0.001	0.982	0.992	0.478–2.057	Not significant
Family members	0.431	4.712	0.030	1.538	1.043–2.269	Significant
Employment status	0.262	3.979	0.046	1.299	1.005–1.680	Significant

## Discussion

The findings of the present study showed that the majority of participants had mixed attitudes toward childhood vaccination, with nearly three‐quarters of respondents falling in the neutral category. Only 8.1% of participants had a strongly positive attitude. However, despite having an overall neutral perception, most participants held positive beliefs about vaccine safety and efficacy. For example, over 60% of parents agreed or strongly agreed that they feel safe after being vaccinated and that vaccines can prevent serious infectious diseases. Similarly, the vast majority rejected conspiracy narratives. Approximately 60-70% of respondents strongly disagreed that vaccines are manipulated for political aims or serve only pharmaceutical profits, and over 70% disagreed that authorities promote vaccination for financial gain. These findings are broadly in line with other recent studies. For example, a cross-sectional study of 233 parents in Saudi Arabia found that although overall awareness and acceptance of vaccination were high, 9% of parents reported delaying vaccination, and a small proportion expressed hesitancy. The main concerns driving hesitancy were fear of serious side effects (51%), concerns about newer vaccines (44%), and worries about administering multiple vaccines at once (44%) [4]. Similarly, another study by Alsubaie et al. reported 20% rate of vaccine hesitancy, based on data from 500 parents. Higher educational level was significantly associated with greater hesitancy (p < 0.001), and among hesitant parents, 36% of children were not fully vaccinated for their age. Safety concerns were the most common reason (53%) for hesitancy. In multivariate analysis, beliefs that vaccines are ineffective (adjusted odds ratio (AOR) = 28, 95% CI = 7.9-102.3) and not important (AOR = 27, 95% CI = 5.8-126) were strongly associated with both parental hesitancy and incomplete child vaccination [15].

Similar evidence was presented in another study from Tabuk, Saudi Arabia. Younis et al. reported that 66.1% of the parents demonstrated adequate knowledge and 55.5% had a positive attitude toward childhood vaccination, while 62.9% adhered to the immunization schedule [16]. Factors significantly associated with better knowledge and attitudes included age 30 to <40 years (OR = 2.6 and 2.3), higher education level (OR = 2.1 and 1.7), and working in the health sector (OR = 2.7 and 4.4), all with p-values <0.05. A strong positive correlation was observed between knowledge and attitude scores (r = 0.603, p < 0.001). Among non-adherent parents, the main barriers were family member illness (36.7%), child illness (32.8%), and being busy on vaccination day (31.4%) [16]. Similar evidence has been reported in international studies. For instance, a study from Malaysia, using the Parent Attitudes About Childhood Vaccines questionnaire, reported that vaccine hesitancy was reported in 11.6% of the parents. Multivariable logistic regression showed that pregnant mothers expecting their first child were significantly more likely to be vaccine-hesitant (AOR = 3.91, 95% CI = 1.74-8.79), and unemployed parents also had higher odds (AOR = 1.97, 95% CI = 1.08-3.59). The internet was the most common source of vaccination information (65.6%), followed by brochures (56.9%) [17]. Similar results were reported in a study from Italy. Giambi et al. surveyed 3,130 parents and reported that 83.7% were pro-vaccine, 15.6% were vaccine-hesitant, and 0.7% were anti-vaccine. Safety concerns were the leading reason for refusal (38.1%) or interruption (42.4%) of vaccination, with hesitant and anti-vaccine parents reporting greater fear of both short-term and long-term adverse effects compared to pro-vaccine parents. Factors significantly associated with hesitancy included lack of pediatrician recommendation, discordant information, and knowing a child with adverse reactions. Despite hesitancy, most parents still recognized the benefits of vaccination and trusted pediatricians as a key information source [18]. This aligns with the findings of the present study, in which 58.5% of participants agreed or strongly agreed that they feel safe after vaccination.

In contrast, in a multi-country Middle East survey on COVID-19 vaccination intentions, only 56% of Arab parents planned to vaccinate their children, with side effects, efficacy, and scheduling cited as key barriers [19]. Although the study focused on COVID‐19 and not routine vaccines, it similarly highlights that safety concerns and vaccine novelty erode parental confidence. Similarly, a study from several European Union countries reported that the vaccine hesitancy rate was 44.3%. They concluded that vaccine hesitancy is not purely country-specific but shared across populations. The findings suggest that differences between countries are less pronounced than expected [20]. Our Riyadh sample’s relatively low outright refusal but large neutral group suggests that many parents are unsure rather than firmly anti-vaccine. Our findings indicate that Riyadh parents broadly trust official vaccination efforts. Conspiracy beliefs were uncommon. Over 70% rejected the notion that vaccines mainly profit pharmaceutical companies in the present study. Alhassan et al. reported that in Saudi society, political trust, loyalty, quality strategic policies, and religious faith have bolstered confidence in vaccine programs [21]. Generally, literature shows that trust in health authorities is a powerful predictor of vaccine acceptance. For example, Liu and Chu found that trust was positively associated with attitude toward the COVID-19 vaccines and vaccination intention, both directly and through positive emotional evaluation of the vaccine [22]. Some other studies center around healthcare trust and misinformation. Janiak et al. found that parents who trust their healthcare provider intend to vaccinate their children [23], while Almuqbil et al. found that parents in Riyadh are hesitant to vaccinate their children because of social media and safety concerns too [24]. At an international level, Lane et al. [25] and Larson et al. [26] studied vaccine hesitancy as a global issue affected by sociocultural, political, and interpersonal factors.

There are several limitations of the study that should be considered while interpreting the study findings. As a convenience online survey, the sample may over-represent urban, educated parents and under-represent rural or less-educated families. The cross-sectional design captures attitudes at one point during evolving political and pandemic dynamics; attitudes may shift as new information or policies emerge. We did not measure trust in government or media directly with validated scales, but the attitude items about political motives serve as indirect indicators. Finally, because the study focused on general childhood vaccines, extrapolation to specific vaccines should be done cautiously.

## Conclusions

This study found that parental attitudes toward childhood vaccination in Riyadh were predominantly neutral, while a smaller proportion of parents expressed negative attitudes and only a limited number demonstrated clearly positive attitudes. These findings suggest that although strong opposition to vaccination was not widespread, many parents remain uncertain and may still be vulnerable to doubts, misinformation, or an incomplete understanding of vaccine benefits. Participants generally acknowledged the role of vaccines in preventing serious infectious diseases and recognized the importance of obtaining information from reliable sources. At the same time, concerns about possible side effects, long-term safety, and beliefs favoring natural immunity were still present. Several sociodemographic factors were associated with more negative attitudes, indicating that parental perceptions are influenced not only by knowledge but also by family and social circumstances. Overall, the study underscores the importance of sustained community engagement, accessible health education, and strong communication from trusted healthcare professionals.
